# Antibiotics for the primary prevention of acute rheumatic fever: a meta-analysis

**DOI:** 10.1186/1471-2261-5-11

**Published:** 2005-05-31

**Authors:** Katharine A Robertson, Jimmy A Volmink, Bongani M Mayosi

**Affiliations:** 1 Primary Health Care Directorate, Faculty of Health Sciences, University of Cape Town, Cape Town, South Africa; 2 The Cardiac Clinic, Department of Medicine, Groote Schuur Hospital and University of Cape Town, Cape Town, South Africa

## Abstract

**Background:**

Rheumatic fever continues to put a significant burden on the health of low socio-economic populations in low and middle-income countries despite the near disappearance of the disease in the developed world over the past century. Antibiotics have long been thought of as an effective method for preventing the onset of acute rheumatic fever following a Group-A streptococcal (GAS) throat infection; however, their use has not been widely adopted in developing countries for the treatment of sore throats. We have used the tools of systematic review and meta-analysis to quantify the effectiveness of antibiotic treatment for sore throat, with symptoms suggestive of group A streptococcal (GAS) infection, for the primary prevention of acute rheumatic fever.

**Methods:**

Trials were identified through a systematic search of titles and abstracts found in the Cochrane Central Register of Controlled Trials (Cochrane Library Issue 4, 2003), MEDLINE (1966–2003), EMBASE (1966–2003), and the reference lists of identified studies. The selection criteria included randomised or quasi-randomised controlled trials comparing the effectiveness of antibiotics versus no antibiotics for the prevention of rheumatic fever in patients presenting with a sore throat, with or without confirmation of GAS infection, and no history of rheumatic fever.

**Results:**

Ten trials (n = 7665) were eligible for inclusion in this review. The methodological quality of the studies, in general, was poor. All of the included trials were conducted during the period of 1950 and 1961 and in 8 of the 10 trials the study population consisted of young adult males living on United States military bases. Fixed effects, meta-analysis revealed an overall protective effect for the use of antibiotics against acute rheumatic fever of 70% (RR = 0.32; 95% CI = 0.21–0.48). The absolute risk reduction was 1.67% with an NNT of 53. When meta-analysis was restricted to include only trials evaluating penicillin, a protective effect of 80% was found (Fixed effect RR = 0.20, 95% CI = 0.11–0.36) with an NNT of 60. The marginal cost of preventing one case of rheumatic fever by a single intramuscular injection of penicillin is approximately US$46 in South Africa.

**Conclusion:**

Antibiotics appear to be effective in reducing the incidence of acute rheumatic fever following an episode of suspected GAS pharyngitis. This effect may be achieved at relatively low cost if a single intramuscular penicillin injection is administered.

## Background

Rheumatic fever is the most common cause of acquired heart disease in children and adults worldwide [1,2]. Acute rheumatic fever is expressed as an inflammatory reaction that involves many organs, primarily the heart, the joints, and the central nervous system [3]. The clinical manifestations of acute rheumatic fever follow a group A streptococcal (GAS) infection of the tonsillopharynx after a latent period of approximately 3 weeks. The major importance of acute rheumatic fever is its ability to cause fibrosis of heart valves, leading to crippling hemodynamics of chronic heart disease, heart failure and death. Open heart surgery may be needed to repair or replace heart valves in patients with severely damaged valves, the cost of which is exorbitant and a drain on the limited health resources of poor countries [4,5].

The global burden of disease caused by rheumatic fever currently falls disproportionately on children living in the developing world. Rheumatic heart valve disease causes 400,000 deaths annually mainly among children and young adults living in developing countries. At least 12 million people are estimated to be currently affected by rheumatic heart disease with two million patients requiring repeated hospitalisation and one million requiring, often unaffordable, heart surgery in the next 5 to 20 years [6]. In many developing countries, the incidence of acute rheumatic fever approaches or exceeds 100 per 100,000, whereas the incidence is currently estimated at less than 2 per 100,000 in the US.[1,2] This decline of rheumatic fever in the industrialised world has been partially attributed to the use of antibiotics for the treatment of GAS infections [7], but it is also believed to be the result of improved living conditions [8]. However, the recent resurgence of rheumatic fever in middle-class families in some parts of the economically developed world is a reminder that even in industrialised countries, there is no room for complacency [9,10].

The bulk of the research on the prevention of rheumatic fever dates back to the mid-20^th ^century and was conducted primarily on adult populations living in the United States [11]. Methods for preventing rheumatic heart disease have included both primary and secondary prevention strategies. Primary prevention is achieved by disrupting the initial transmission of GAS infection or by blocking the progression of GAS infection to rheumatic fever. Secondary prevention is used following an attack of acute rheumatic fever to prevent the progression to cardiac disease. The consensus reached in the 1950's was that the most effective and efficient method for preventing rheumatic heart disease was through the primary prevention of acute rheumatic fever using antibiotics to treat the preceding GAS infection (WHO 1954) [12]. Where primary prevention failed, a secondary prevention plan also relying on antibiotic therapy was recommended for preventing the progression of cardiovascular complications [13].

Treatment of streptococcal pharyngitis with antibiotics is currently standard practice in most of the developed world. To note some exceptions, there are a few countries that now recommend no investigation or treatment of sore throat based on recent fears of increasing antibiotic resistance compounded with the findings of a recent study highlighting the negligible benefits gained from antibiotic treatment [14]. This new rationale for treating sore throat with antibiotics may not be applicable to a developing country setting where there remains a real threat of rheumatic fever. Antibiotic treatment of streptococcal pharyngitis appears to be an effective health intervention that is simple to administer; however, its benefits have not been realised in much of the developing world [1].

Controversy exists over the priority that the primary prevention of rheumatic fever deserves in the competition for scarce resources for healthcare in developing countries [1]. In this context it has been argued that secondary rather than primary prevention should be the mainstay of community-based approaches to the control of rheumatic fever and rheumatic heart disease [1]. Unfortunately, these discussions take place in an information vacuum, since the benefits of primary prevention of rheumatic fever, to the best of our knowledge, have not been quantified in a systematic fashion. This review aims to summarise the evidence on the effectiveness of antibiotics for the prevention of acute rheumatic fever, and seeks to determine to what extent the introduction of primary prevention programmes for rheumatic fever is supported by existing research.

## Methods

### Study search

We systematically searched the Cochrane Central Register of Controlled Trials (Cochrane Library Issue 4, 2003), MEDLINE (1966–2003), EMBASE (1966–2003), and the reference lists of identified studies. Search terms included: sore throat, pharyngitis, rheumatic, streptococcal pharyngitis, strep throat, antibiotics, and tonsillitis. We reviewed the selected titles and abstracts (when available) to identify which studies were trials. There were no language restrictions.

### Inclusion criteria

We included only randomised and quasi-randomised controlled trials on a patient population presenting with a sore throat (pharyngitis) with or without confirmation of GAS infection by a throat culture and/or a rapid test, and no history of rheumatic fever. Trials that did not specify the method of randomisation and for which the authors were not available for follow-up were excluded.

Trials were required to use the Jones Criteria for the diagnosis of rheumatic fever. A positive diagnosis required the presence of 2 major criteria [carditis, migrating polyarthritis of the big joints, chorea, erythema marginatum, subcutaneous nodules] or 1 major and 2 minor criteria [fever, arthralgia without arthritis, previous history of RF or RHD, prolonged P-R interval, elevated erythrocyte sedimentation rate, positive C-reactive protein, leucocytosis] [15].

Acceptable interventions included any antibiotic versus placebo or no treatment. Acceptable trials included the incidence of a first attack of acute rheumatic fever following throat infection as an outcome. Secondary outcomes that were noted but not required included adverse events to antibiotic use, adherence to antibiotic therapy and mortality associated with the first attack of rheumatic fever.

### Data extraction

Using a standardized data form, two reviewers (KR, BM) independently extracted data from each eligible trial. The methodological quality of the trials was assessed based on the method of randomisation, concealment of allocation, blinding and loss to follow-up. Additional data extracted from each trial included information on the study setting (i.e.: hospital vs. community-based trials, geographic location), on the participants (age, presenting complaint), and on the details of the interventions.

### Data analysis

The results from each study were summarised using relative risk and a 95% confidence interval (CI). The chi-squared test for heterogeneity was used, along with the visual inspection of graphs to determine the level of between study variation. As the p-value associated with this test was large (>0.10), indicating low-levels of statistical heterogeneity between study results, a fixed-effect model was used to pool the data to estimate an overall effect. A subgroup analysis included only studies where penicillin was used as the intervention.

## Results

### Study search

**Figure 1:**
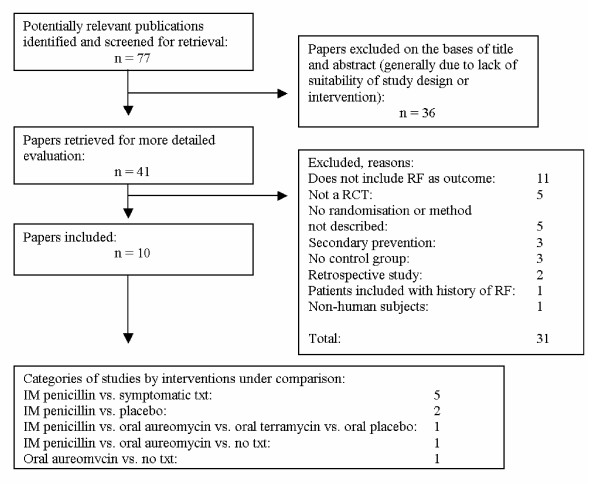
The QUORUM statement.

Seventy seven potentially eligible studies were identified by the preliminary search. Based on their titles and abstracts (when available), 41 studies were retrieved for a thorough evaluation, performed independently by two reviewers (KAR, BMM) (Fig. 1). Discrepancies in the selection of studies were resolved through discussion. The reviewers were not blinded to the source, institution or results of the studies.

Thirty one of 41 studies that were retrieved in full-text were excluded for the following reasons: rheumatic fever not included as an outcome (11), not trials (5), no randomisation or method of randomisation not described (5), trial on secondary prevention of rheumatic fever (3), control group not used (3), retrospective study (2), patients included with a history of rheumatic fever (1), non-human subjects used (1).

### Characteristics of included studies

Ten studies were included in the review (Table 1) [16-25]. The studies were all hospital-based, 8 out of 10 of which were conducted at U.S. military hospitals from 1950 to 1957 [16,18-24]. Only 3 of the studies used placebos for the control group [20,21,23]. The remainder used either no treatment or symptomatic treatment as controls. Patients in the US-based studies comprised males, aged 17 years and older [16,18-24]. One trial included children aged 3–16 [25], and another involved adults and children [17]. We sought only studies on patients with no previous history of rheumatic fever. While 3 of the 10 included studies reported some patients with a rheumatic fever history [16,19,22], the rates were below the margin of error (5.0%) and therefore negligible. All 10 studies made the attempt to limit the study participants to those with suspected GAS infection, a diagnosis that was based on the observance of exudate on the tonsils or pharynx in 9 out of 10 studies. The use of this single criterion for the diagnosis of GAS infection was supported by a study estimating that 70–90% of patients admitted to army hospitals around the same time period with streptococcal tonsillitis or pharyngitis presented with exudate on their tonsils or oropharynx [26].

**Table 1:** Characteristics of included trials

**Study ID**	**Randomisation and concealment of treatment allocation**	**Participants**	**Intervention**	**Effect size**	**% Randomised included in analysis**
Bennike, 1951	Quasi-randomised; Inadequate concealment of treatment	349 admitted to hospital with ordinary acute tonsillitis, plegmonous tonsillitis or ulcerative tonsillitis	1. Penicillin: IM, 300,000 units/day for 6 days (adults) 2. Control – symptomatic treatment	Not Estimable	88%
Brink, 1951	Quasi-randomised; No concealment of treatment	475 males, aged 17–21, admitted to U.S. military hospital with respiratory symptoms or fever with exudate on tonsils or pharyngeal mucosa	1. Procaine penicillin G: IM 300,000 units/day for 4 days 2. Aureomycin: avg. 2 g/day orally for 4 days 3. Control – no treatment	RR = 0.29 [0.06,1.46]	Unknown
Brock, 1953	Randomised; No concealment of treatment	349 males admitted to U.S. military hospital with exudative pharyngitis and laboratory-confirmed GAS infection	1. Procaine penicillin G: IM 600,000 units/day for 3 days 2. Control: IM saline placebo, day 1 and day 5	RR = 0.11 [0.00,2.71]	Unknown
Brumfitt, 1957	Quasi-randomised; No concealment of treatment	121 males, aged 18–21, admitted to U.S. military hospital with sore throat, pyrexia and no clinical evidence of more generalized disease of which sore throat may have been coincident feature	1. Combination of procaine penicillin G: IM 600,000 units/day for 4 days and crystalline penicillin: IM 200,000 units/day for 4 days 2. Control – symptomatic treatment	Not estimable	Unknown
Chamovitz, 1954	Quasi-randomised; Unknown whether treatment allocation concealed	241 males admitted to U.S. military hospital with exudative tonsillitis or pharyngitis	1. DBED penicillin: IM 1,200,000 units 2. Control – IM placebo	RR = 0.17 [0.01,3.41]	Unknown
Denny, 1950	Quasi-randomised; No concealment of treatment	1602 males admitted to U.S. military hospital with respiratory symptoms and observed exudate on the tonsils or pharyngeal wall	1. Penicillin G: IM 200,000 units/day for 3 days of 300,000 units/day for 4 days 2. Control: symptomatic treatment	RR = 0.12 [0.03,0.50]	81.8%
Denny, 1953	Randomised; Unknown whether treatment allocation concealed	207 males admitted to U.S. military hospital with suspected streptococcal infection based on presence of exudate on tonsils or pharynx and total leukocyte count exceeding 10,000	1. Crystalline procaine penicillin: IM 600,000 units/day for 5 days 2. Crystalline aureomycin: avg. 2 g/day for 5 days 3. Crystalline terramycin: avg. 2 g/day for 5 days 4. Control: oral lactose placebo for 5 days	RR = 0.64 [0.06,6.92]	Unknown
Houser, 1953	Quasi-randomised; No concealment of treatment	2044 males, ages 17–21, admitted to U.S. military hospital with exudative lesions on their tonsils or pharynx	1. Aureomycin: avg. 2 g/day for avg. 5 days 2. Control: no specific treatment	RR = 0.63 [0.34,1.17]	88%
Siegel, 1961	Quasi-randomised; No concealment of treatment	1213 children, aged 3–16, with uncomplicated acute upper-respiratory tract disease and laboratory-confirmed GAS infection	1. Benzathine penicillin G: IM 600,000 units 2. Control: symptomatic treatment	RR = 0.20 [0.01,4.14]	95%
Wannamaker, 1951	Quasi-randomised; No concealment of treatment	2340 males, aged 17–20, admitted to U.S. military hospital with respiratory symptoms and exudative lesions on the tonsils or oropharynx, or oral temp. > 100°F	1. Procaine penicillin G: IM various dosages (1,200,000 over 4 days; 600,000 units over 3 days; 600,000 single dose) 2. Control: no specific treatment	RR = 0.21 [0.09,0.47]	83.3%

All 10 studies conducted appropriately timed follow-up visits at 3–4 weeks following the initial onset of symptoms given the 3-week delay in the onset of clinical signs of rheumatic fever. Adherence to therapy was not measured in any of the studies, as all of the studies included were hospital-based and antibiotics were not self-administered. Approximately half of the studies mentioned side effects as an outcome; however, most were not quantified. Symptoms mentioned were pain at the site of injection, nausea and diarrhea.

The methodological quality of the studies, in general, was poor. Nine out of the ten studies reviewed date back to the 1950's when randomised trial methods were still evolving and good guidelines for conducting trials were not yet available [16-24]. Regarding randomisation and allocation concealment, two trials used shuffled cards and were considered to be truly randomised [20,21]. The remaining studies were categorized as 'quasi-randomised' as allocation was based on methods such as Air Force Serial number, and date of hospital admission. As an "open" system of randomisation was used in all trials, allocation concealment was deemed inadequate in all cases. Three out of 10 studies were placebo-controlled [20,22,23], however, this did not ensure blinding of patients and providers as the placebo used was not identical in appearance to the experimental treatment. Four studies employed blinded assessors during follow-up for the diagnosis of rheumatic fever [16,18,19,22], 2 did not specify whether the assessor was blinded [24,25], and 1 used no blinding [17]. Five out of 10 studies reported on loss to follow-up [16,17,19,22,25]. Data from these trials on the number of patients that were randomised which could be included in the final analysis ranges from 81.8% to 95%.

### Quantitative data synthesis

#### 1. Incidence of rheumatic fever

##### Antibiotics versus control

**Figure 2:**
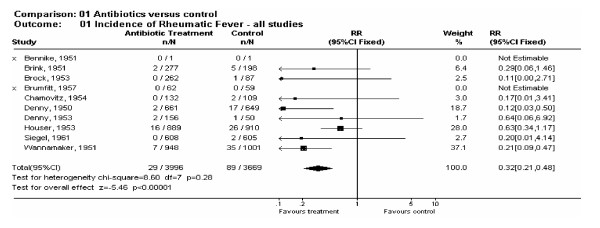
Forest plot of antibiotic trial effect sizes.

All 10 studies evaluated the effectiveness of antibiotics in preventing acute rheumatic fever (n = 7665). Three thousand nine hundred and ninety six (3996) received antibiotic treatment and 3669 received either placebo or no specific treatment. As no statistical heterogeneity was present (p = 0.28) we pooled the results of the individual studies using the fixed-effect model. Meta-analysis revealed a substantial protective effect against the onset of acute rheumatic fever in patients treated with antibiotics (RR = 0.32; 95% CI = 0.21–0.48) (Fig. 2). Twenty nine of 3996 (0.73%) patients taking an antibiotic, and 89 of 3669 (2.4%) patients receiving no antibiotic developed acute rheumatic fever 1–2 months following a suspected streptococcal sore throat infection. These findings suggest that administering antibiotics to a patient with a sore throat and symptoms suggestive of GAS infection, who has no history of rheumatic fever, will reduce his or her risk of acute rheumatic fever by almost 70%. The number of patients with suspected GAS throat infection needed to treat with antibiotics to prevent 1 case of acute rheumatic fever is 53 (NNT = 53).

##### Sub-group analysis – intramuscular penicillin versus control

**Figure 3:**
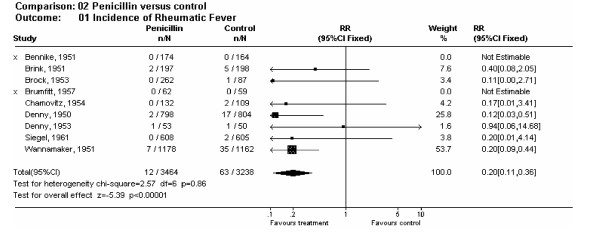
Forest plot of penicillin trial sizes.

Nine out of ten studies compared the effect of intramuscular penicillin versus control for the prevention of acute rheumatic fever [16-21,23-25]. We found no statistical heterogeneity (p = 0.86) and conducted a fixed-effect, meta-analysis revealing an even greater protective effect against acute rheumatic fever with penicillin than all antibiotics combined (RR = 0.20, 95% CI = 0.11–0.36) (Fig. 3). Twelve of 3464 patients (0.35%) treated with penicillin, and 63 of 3238 (2.0%) patients on no antibiotic developed acute rheumatic fever 1–2 months following a suspected streptococcal sore throat infection. These results indicate an 80% reduction in risk of acute rheumatic fever for patients with sore throat and symptoms suggestive of GAS infection when treated intramuscularly with penicillin. This sub-group analysis yielded an NNT of 60.

#### 2. Adverse outcomes

Only 4 out of 10 studies reported any adverse outcomes associated with antibiotic treatment [17,18,20,23]. One study reported on pain at the site of injection [23], and the others reported on nausea, vomiting, and diarrhea following antibiotic treatments. No study reported mortality associated with an attack of acute rheumatic fever (Table 1).

## Discussion

This systematic review found that antibiotic treatment of sore throat with accompanying symptoms suggestive of group A streptococcal (GAS) infection is effective in reducing the attack rate of acute rheumatic fever by 70%. Intramuscular penicillin appears to reduce the attack rate by as much as 80%. There was one fewer case of acute rheumatic fever for every 50–60 patients treated with antibiotics. These findings suggest that antibiotic treatment can be effective for preventing acute rheumatic fever in a population with suspected GAS throat infection.

The treatment of GAS pharyngitis is directed towards eradication of the bacteria from the upper respiratory tract. The infection can usually be eradicated by a single intramuscular injection of benzathine benzylpenicillin or by 10 days' treatment with oral penicillin [1]. While the use of intramuscular penicillin is supported by clinical trials, few trials have been done to test the efficacy of the oral penicillin for preventing acute rheumatic fever [27]. There is resistance to using intramuscular penicillin in some developing countries due to the perceived higher risk of anaphylaxis, the dangers associated with the potential reuse of needles and the discomfort of intramuscular injections. Concerns over safety issues have resulted in government orders prohibiting penicillin injections in hospitals and clinics [28]. Government regulations in response to some of these fears are warranted, particularly in the area of infection control through the prevention of needle reuse. However, with respect to the dangers of anaphylaxis, more than 50 years of experience with penicillin has shown that, while toxic reactions to intramuscular penicillin have been reported, severe reactions are exceedingly rare, especially in children. Therefore, when given under sterile conditions, fear regarding the use of parenteral penicillin is unwarranted [1].

The challenge for policymakers and clinicians is how best to apply these findings to a developing country setting where the risk of rheumatic fever persists. The most evident hindrance to the generalisability of these findings is the discrepancy in characteristics of the studied population and the population currently at risk for rheumatic fever. All of the included studies were conducted in a developed country setting a half century ago, and 8 out of 10 studies included only young adult males in their study population. Variables such as geographic location and age will affect the incidence of GAS infection as well as the attack rate of acute rheumatic fever within a population. These epidemiological differences across populations will in turn affect the number of cases needed to treat (NNT) with antibiotics in order to prevent one case of rheumatic fever. Unfortunately, trials similar to those included in this review have not been conducted in developing countries. However, the strong findings of this review do provide supportive evidence for the development of primary prevention programs to decrease the long-term sequelae associated with GAS pharyngitis in developing countries.

The validity of applying the NNT found in this review to pediatric populations in developing countries is questionable due to probable differences in GAS infection rates. In populations with a high incidence of GAS infection, the proportion of patients with respiratory symptoms that are infected with GAS will be higher than in a setting of low GAS infection incidence. A higher GAS incidence will yield a lower NNT, under the assumption that antibiotics provide the same protective effect in both populations. In the population studied, the proportion of suspected GAS infections that were true positives was large given the high specificity of the presence of exudate in the oropharynx area, estimated at 70–90% [26]. To improve the generalisability of these results to the present developing country setting, criteria for identifying suspected GAS would need to exhibit similar levels of specificity.

Identifying true cases of streptococcal pharyngitis is difficult in a developing country setting where laboratory confirmation of GAS infection is not readily available. Therefore there is a need to use a clinical prediction rule (CPR) for the diagnosis of GAS infection that takes into account these limitations while at the same time maximizing both sensitivity and specificity. Several CPR's have been developed and implemented worldwide for diagnosing streptococcal pharyngitis. A CPR put forth by the WHO Acute Respiratory Infection (ARI) Guideline suggests that both pharyngeal exudate and an enlarged and tender cervical node should be present [29]. An evaluation of this CPR in a developing country setting estimated the sensitivity and specificity of these criteria at 12% and 94% respectively [30]. The high specificity ensures that suspected GAS cases have a high probability of being true cases; however, the low sensitivity suggests that many true cases are not identified. It is at this point that a CPR suitable for a developed country diverges from a CPR suitable for a developing country where the risk of acute rheumatic fever is significantly higher.

In developing countries, it is more important to detect and treat all possible cases of GAS infection than to prevent antibiotic treatment for sore throats not attributable to GAS infection. Therefore an appropriate CPR for this setting will have high sensitivity, despite the subsequent tradeoffs of a lowered specificity. It was found that a revised version of the WHO CPR that included either the presence of exudate or enlarged cervical nodes exhibited a sensitivity and specificity of 84% and 40% respectively when tested in a developing country setting [30]. It should be noted that the loss of specificity will lead to an increase in the NNT. The tradeoffs that exist between a CPR's sensitivity, specificity, and the resulting NNT have implications for the cost of implementing a primary prevention program for rheumatic fever.

In the absence of accurate epidemiological data on streptococcal pharyngitis and rheumatic fever for the developing world, a formal cost-effectiveness analysis of the use of antibiotics for the primary prevention of rheumatic fever is not feasible. However, anecdotal evidence exists supporting the cost-effectiveness argument. The cost of one intramuscular injection of penicillin (1.2 million units) in South Africa is ~ R5.00. Using the NNT found in this review, one case of rheumatic fever will be prevented for every 60 patients receiving one intramuscular injection of penicillin for the treatment of suspected streptococcal pharyngitis. Therefore the marginal cost of preventing one case of rheumatic fever is R300.00, or US$46. A recent study in Sao Paulo, Brazil estimates the economic burden associated with rheumatic fever and its long-term sequelae in a low-income population to be over US$50 million. Furthermore, treatment costs for chronic rheumatic patients were estimated at US$319/patient/year [31]. Comparing these costs with the estimated costs of prevention provides support for the cost-effectiveness of intramuscular antibiotic treatment. An accurate cost-effectiveness analysis can not yet be done for oral antibiotics until more conclusive evidence on its efficacy is available.

Intuitively, primary prevention is a more desirable outcome for the individual as well as the health sector. Primary prevention decreases the burden placed on patients and health facilities by decreasing reliance on time- and resource-intensive secondary prevention programs, the success of which is entirely dependent on patient compliance. However, secondary prevention programmes are currently thought to be a more cost-effective approach to RF/RHD prevention and therefore more deserving of scarce resources available to communities in poor countries. This study highlights the lack of reliable data required to accurately assess the cost-effectiveness of a primary prevention program. While there is some scattered data on the prevalence of RHD in developing countries, additional data on the incidence of RF in these settings as well as the incidence of GAS infections is still needed. Furthermore, the preliminary cost-analysis put forth by this study suggesting that primary prevention is likely to be cost-effective, when considered in light of the favorable outcome of primary prevention, calls for additional attention and evaluation. At this point, primary prevention should not be dismissed.

It is important to note that the results of the included trials could have been distorted by either selection bias, detection bias, or both, arising from poor methodological quality. Few studies were adequately randomised, none used an appropriate method for concealing allocation, only four blinded the outcome assessors, and only five commented on subjects lost to follow-up. Empirical evidence suggests that trials with inadequate or unclear allocation concealment or lack of blinding, on average, lead to overestimation of the effects of interventions [32].

## Conclusion

Acute rheumatic fever is common among children living in poor socioeconomic conditions. The findings of this review support the notion that antibiotic treatment given to cases of suspected streptococcal pharyngitis is an effective and safe option for reducing the complication of acute rheumatic fever. In other parts of the world the incidence of acute rheumatic fever is so low that the risks of antibiotic use may outweigh the potential benefits. Evidence is needed concerning the effects of antibiotic therapy for preventing rheumatic fever and rheumatic heart disease in children living in developing countries. Given the overwhelmingly positive results in the trials included in this review, placebo-controlled trials could be considered unethical. Trials comparing antibiotic types, routes of administration, and treatment regimens within high-risk groups, however, should be considered. Additionally, the collection of baseline epidemiological data on GAS infections, rheumatic fever, and rheumatic heart disease, and compliance with existing guidelines [33] is needed at the country level. Data on the ratio of symptomatic to sub-clinical cases of GAS pharyngitis is also essential for health planners to assess the level of prevention that is obtainable through primary prevention. Together, these data will enable countries to make more accurate estimates of the NNT and to assess the potential impact of both primary and secondary prevention programs on the health and economic outcomes associated with rheumatic fever.

## Competing interests

The author(s) declare that they have no competing interests.

## Authors' contributions

KAR and BMM contributed to all aspects of this study. JAV contributed to study design, data analysis and writing of the paper. All authors read and approved the final manuscript.

## Pre-publication history

The pre-publication history for this paper can be accessed here:


